# Role of Specialized Pro-Resolving Mediators in Modifying Host Defense and Decreasing Bacterial Virulence

**DOI:** 10.3390/molecules26226970

**Published:** 2021-11-18

**Authors:** Julianne M. Thornton, Kingsley Yin

**Affiliations:** Department of Cell Biology and Neuroscience, Rowan University School of Osteopathic Medicine, Stratford, NJ 08084, USA; thorntonj4@rowan.edu

**Keywords:** resolvins, lipoxin, quorum sensing, innate immunity, neutrophils, macrophages

## Abstract

Bacterial infection activates the innate immune system as part of the host’s defense against invading pathogens. Host response to bacterial pathogens includes leukocyte activation, inflammatory mediator release, phagocytosis, and killing of bacteria. An appropriate host response requires resolution. The resolution phase involves attenuation of neutrophil migration, neutrophil apoptosis, macrophage recruitment, increased phagocytosis, efferocytosis of apoptotic neutrophils, and tissue repair. Specialized Pro-resolving Mediators (SPMs) are bioactive fatty acids that were shown to be highly effective in promoting resolution of infectious inflammation and survival in several models of infection. In this review, we provide insight into the role of SPMs in active host defense mechanisms for bacterial clearance including a new mechanism of action in which an SPM acts directly to reduce bacterial virulence.

## 1. Introduction

During bacterial infection, host defense is activated. The earliest part of host defense, the innate immune system, comprises: (i) physical barriers such as tight junctions, mucous membranes, epithelial cells, endothelial cells, and smooth muscle, (ii) immune cells, and (iii) complement formation. This innate immune system is the first line of host defense critical for pathogen clearance. Adaptive immunity is involved in the later stage clearance of pathogen and for memory. With respect to innate immunity, there are three processes that are particularly important: (1) phagocytosis, (2) inflammation, and (3) production of antimicrobial molecules or traps, namely, defensins, free radicals, and neutrophil extracellular traps. In this review, we will focus specifically on the innate immune cell component of host defense to bacterial infection.

Infectious activation of innate immunity occurs when biological molecules termed Pathogen Associated Molecular Patterns (PAMPs) bind to Pattern Recognition Receptors (PRRs), of which the Toll-Like Receptor systems (TLRs) are the most studied. In particular, TLRs 2 and 4 respond to binding of PAMPs such as lipopolysaccharide (LPS) and lipoteichoic acid (LTA) [1,2,3,4,5]. These receptors are widely expressed on cells of innate immunity (neutrophils, monocytes, macrophages, NK cells, dendritic cells, T and B cells) as well as nonimmune cells (epithelial cells, endothelial cells, fibroblasts) [4,5]. Binding of TLRs activates intracellular signaling cascades that result in the production of defensins, increased phagocytosis and inflammatory mediators such as cytokines, chemokines, arachidonic acid metabolites and free radicals [6,7]. The production of defensins, formation of reactive oxygen species, extracellular traps and phagocytosis are processes that are directly involved in killing the bacterial pathogen. The inflammatory response is also essential for pathogen clearance. At the cellular level, the inflammatory response helps leukocytes move along blood vessels, adhere to the endothelium, and cross the endothelial barrier to reach the site of infection. Clinically, the inflammatory response causes raised body temperature, pain, redness, and edema. It is now believed that this acute inflammatory response requires an active, coordinated resolution for the host to achieve homeostasis and prevent chronic inflammation [8,9]. Failure to resolve acute inflammation, which is a result of sustained infection (particularly in bacterial infection), leads to initial hyperinflammation with tissue injury, followed later by immunosuppression where the host is unable to clear the preexisting infection or has increased susceptibility to secondary opportunistic infections. This immunosuppression is characterized by (i) reduced lymphocyte numbers, (ii) decreased macrophage function (reduced phagocytic ability, decreased response to LPS stimulation), and (iii) attenuated neutrophil migration [1,10]. Although there is a major difference in the pathophysiological presentation of chronic inflammation versus chronic infection in that there must be an invading pathogen to be termed “infection”, the “solution” to the problem is similar: active resolution of the process (inflammation or infection) and return to homeostasis.

Resolution of bacterial infection involves (i) clearance of bacterial load, (ii) inhibition of neutrophil sequestration and increased neutrophil apoptosis, (iii) reduction in systemic proinflammatory response, (iv) increased macrophage recruitment, and (v) augmentation of macrophage phagocytosis and efferocytosis of apoptotic neutrophils [11,12]. These processes are essential for returning the host to homeostasis. Therefore, an unanswered paradox is: do resolution mediators, which typically reduce inflammation and neutrophil function, also attenuate host defense in general? If so, which parts of host defense? This review will examine the pertinent literature on a specific group of bioactive fatty acids—the Specialized Proresolving Mediators (SPMs) to provide insight into their role in host defense during bacterial infection.

## 2. Specialized Proresolving Mediators (SPMs)

**Figure 1:**
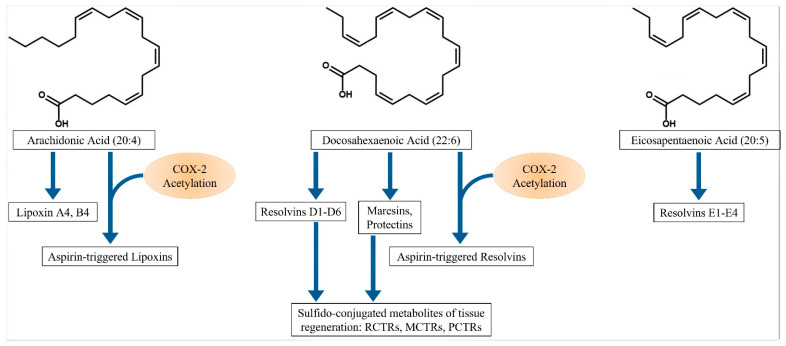
Specialized Proresolving Mediators (SPMs) are produced from arachidonic acid (20:4), docosahexaenoic acid (22:6), and eicosapentaenoic acid (20:5).

Serhan and colleagues isolated and elucidated the structure of a group of novel lipid compounds, the SPMs, which have strong inflammation resolution activity [8,9]. The SPMs are formed from arachidonic acid (20:4), eicosapentaenoic acid (20:5), docosahexaenoic acid (22:6), and docosapentaenoic acid (22:5) (Figure 1), and they exert their resolution actions through binding of specific receptors [8,9,13,14,15,16]. These compounds are produced by transcellular synthetic pathways involving various cell types, such as macrophages, neutrophils, platelets, epithelial cells, and endothelial cells.

Briefly, arachidonic acid can be converted to Lipoxins (Lx) through the actions of several lipoxygenase (LOX) enzymes on different cell types. For instance, neutrophil-derived 5-LOX and either platelet-derived 12-LOX or monocyte/macrophage-derived 15-LOX can form LxA4. Aspirin-triggered LxA4 (AT-LxA4) also known as 15-epi-LxA4 is made from the acetylation of the cyclooxygenase-2 (COX-2) enzyme by aspirin. This acetylation of COX-2 directs the enzyme to form 15(*R*)-hydroxyeicosatetraenoic acid (15(*R*)-HETE) from arachidonic acid. 5-LOX then converts the 15(*R*)-HETE to 15-epi-LxA4 [17]. D-series resolvins (Rvs) are formed from docosahexaenoic acid (DHA). 15-LOX converts DHA to 17(*S*)-hydroperoxyDHA (17(*S*)-HpDHA), which then can be converted by 5-LOX to Rvs D1, D2, D3, D4, D5 and D6. Similar to AT-LxA4, aspirin acetylates COX-2, which then acts on DHA to form the 15-epi-RvDs [18]. Lx compounds, Rvs D1 and D3 bind to Formyl Peptide Receptor-2 (FPR2) and G-Protein coupled Receptor (GPR)-32 to exert their actions [19]. RvD5 also uses GPR32. RvD2, on the other hand, signals through GPR18 [19]. 17(S)-HpDHA can also be converted to Protectin D1 (PD1), while 14(S)-DHA formed from the action of 12-LOX on DHA, can give rise to Maresin 1 (Mar1). PD1 is a ligand for GPR37 [20], while Mar1 signals through Leucine-rich repeat G-protein coupled receptor-6 (LgR6) [21]. E-series Rvs are formed from EPA in a similar fashion as Rvs of the D-series. Thus, 15-LOX converts EPA to 17(*S*)-HpEPA, which then can be acted upon by 5-LOX to make Rvs of the E-series. Similarly, Aspirin-triggered 15-epi-RvEs are formed after acetylated COX-2 acts on EPA. Rvs E1 and E2 are ligands for ChemR23 receptors [19]. RvE1 also binds and inhibits leukotriene B4 receptor-1 (BLT1) [19].

More recently, a novel set of sulfido-conjugated metabolites derived from DHA were elucidated [22,23,24]. These compounds can be formed from epoxide intermediates of Maresin, Resolvin and Protectin and are termed Maresin Conjugates of Tissue Regeneration-1 (MCTR1), Resolvin Conjugates of Tissue Regenaration-1 (RCTR1) and Protectin Conjugates of Tissue Regeneration-1 (PCTR1). All of these compounds can then be converted to the 2 and 3 series metabolites by glutathione *S*-transferase, forming RCTR2, RCTR3, MCTR2, MCTR3, PCTR2, and PCTR3.

There are also resolvins formed from docosapentaenoic acid (22:5; DPA) [15]. These resolvins, termed RvTs, are generated after neutrophil and endothelial cell interactions in much the same way as the resolvins are formed from DHA and EPA. These reports provide strong evidence that SPMs are formed from intercellular interactions.

## 3. SPM Actions in Infectious Inflammation Resolution

The inflammation resolution mechanisms include decreased neutrophil migration, increased neutrophil apoptosis, macrophage recruitment, efferocytosis of apoptotic neutrophils, increased macrophage phagocytic ability, reduced inflammatory mediator and free radical release, and tissue repair [7,8,9,13,14,17,25,26,27,28,29,30]. These actions are critical to return the host to homeostasis. Specifically, increased recruitment of macrophages, a professional phagocyte, promotes bacterial clearance. By the same token, increased neutrophil apoptosis after bacterial phagocytosis, together with efferocytosis by macrophages, also enhances bacterial clearance [17,25,26,27,28,29,30]. Reduction in inflammatory mediator and free radical release helps to attenuate oxidative stress and tissue injury [16]. As inflammatory responses also occur in infection (infectious inflammation), it is reasonable to assume that the beneficial effects of SPMs in infection may also be attributed to their proresolution actions. The question is whether SPMs, in carrying out certain proresolution actions, also affect host defense, i.e., do SPM actions to reduce neutrophil migration, increase neutrophil apoptosis, and attenuate the inflammatory response impede the hosts’ initial ability to fight infection?

## 4. SPM Levels in Bacterial Infections

There is evidence that SPM levels are altered in various bacterial infection models. In a nonhuman primate model of *Streptococcal pneumonia* induced pulmonary infection, blood levels of RvE1 were significantly lowered [31]. Similarly, RvD1 levels were decreased in a rodent model of *Pseudomonas aeruginosa* induced pneumonia [32], suggesting that reduced SPM formation is implicated in pathogenesis of disease.

All RCTRs can be found in human spleens and are present at elevated levels after *Staphylococcus aureus* administration [22]. Human macrophages, when stimulated with *Escherichia coli*, also show an increased production of MCTR1 and MCTR2 [23]. Specific populations of macrophages, the “anti-inflammatory” M2 phenotype, produce resolvins and maresins when stimulated with *E. coli* or *S. aureus* [33]. Similarly, peritoneal exudates taken from mice given *E. coli* showed an increased production of RvD5 and PD1 [34]. Germ-free mice had greater colon levels of RvD1 and RvD5 [34]. Plasma taken from self-resolving infection in mice administered *E.coli* showed a time-dependent rise in D-series Rvs (D1, D2) from 5–8 pg/mL to 20–28 pg/mL in 12 h. RvTs rose from 6–10 pg/mL to 30–50 pg/mL within 4 h [15]. Taken together, the results suggest that SPM production is increased during infection and that this increased production may be an important component of host defense for bacterial clearance and infection resolution.

Dalli et al., 2016 [15] reported that levels of Rvs of the D and E-series, as well as LxA4, were significantly increased by 3- to 10-fold in plasma of septic patients. On the other hand, blood taken at days 1, 3, and 7 from sepsis patients showed increased levels of specific SPMs—RvE1, RvD1, RvD5, and PD1 in nonsurvivors versus survivors [35]. The authors attribute this increase to a “failure to resolve” in the non-survivors.

In early reports, Mori’s group showed that dietary fish oil supplementation (2.4 g/day; 1.4 g EPA, 1 g DHA) for 21 days (long-term) significantly increased plasma levels of 17-hydroxy-DHA (17-HDHA, precursor of D-series resolvins), 18-hydroxy-eicosapentaenoic acid (18-HEPE, precursor of E-series resolvins), RvD1, RvD2 and RvE1 to approximately 0.1–0.5 nM [36]. Shorter term feeding (5 days) was similar but did not raise RvD1 or RvD2. These early reports were supported by more recent work showing that fish oil supplementation (3.4 g/day) for 8 weeks, followed by endotoxin challenge, raised total plasma SPM levels to 3–4 nM [37]. This provides evidence that SPM levels could be raised by dietary precursors of SPMs. In cases of acute bacterial infections, fish oil supplementation apparently may not be particularly efficacious as a therapeutic modality, as it requires weeks to increase SPM levels. Using pure SPMs may be more appropriate in the setting of acute bacterial infections, as it will increase SPM levels quickly. It is possible to speculate that dosing in the high nM range may be of some value. The SPMs that should be considered are RvD2 and LxA4, as these two were shown to be effective in preclinical rodent models [38,39], and they were not shown to correlate with nonsurvivors of sepsis [35].

The results from these independently conducted studies provide evidence that endogenously produced SPMs are implicated in the pathophysiology of bacterial infection. The results also suggest that specific SPMs could be biomarkers of disease severity. SPMs were measured in the range of pg (low nM), which is the bioactive range of these compounds [7].

## 5. SPM-Mediated Effects on Neutrophil Activity

Neutrophils are early cellular responders to infection. Responding to various chemotactic signals, neutrophils migrate to the site of infection, where they attack the invading pathogens by several broad mechanisms: phagocytosis, production of reactive oxygen species, complement and neutrophil extracellular traps (NETs) [11,40,41]. These activities of host defense are essential for bacterial clearance, but sustained neutrophil activation with the release of cytokines and free radicals in this manner can result in tissue injury and organ damage [40,42,43,44].

On the other hand, in severe sepsis where there is overwhelming inflammation and bacteremia, neutrophil dysregulation occurs. In this “dysregulation”, neutrophils fail to respond to chemoattractants, do not migrate to the site of infection, have lowered apoptosis, and can suppress immune function [45,46,47,48,49,50,51]. Interestingly, it was recently reported that bacterial and mitochondrial DNA impaired neutrophil phagocytic ability in lungs of mice administered *P. aeruginosa* [52], suggesting that bacterial components directly impair neutrophil function. This dysregulation can lead to the host’s inability to clear existing infection and can increase susceptibility to opportunistic infections. This paradoxical, deregulatory effect on neutrophil function highlights the importance of resolution of infection and precise modulation of host defense.

SPMs inhibit neutrophil transmigration across the vascular compartment, increase neutrophil apoptosis, and increase phagocytosis [52,53,54,55,56,57]. RvE1 reduced neutrophil number and bacteria load in *E. coli* infected mouse lungs [58]. Similarly, Aspirin-triggered RvD1 increased the rate of neutrophil clearance as well as gram-negative bacterial clearance in infected lungs [59]. Use of an FPR2 antagonist, which is the common receptor for LxA4 and RvD1, impaired bacterial clearance and lung injury in a *Streptococcal pneumoniae* mouse model [60]. These studies are consistent with the postulate that SPMs promote host defense to clear pathogen.

There is evidence that supports the notion that the decreased neutrophil number and/or increased neutrophil clearance is due to neutrophil apoptosis and macrophage efferocytosis of dead neutrophils [26,55,61]. How do decreases in neutrophil migration to infected sites correlate or effect a reduction in bacterial burden? One possible mechanism is through potentiation of neutrophil phagocytic ability. Indeed, LxA4 was shown to increase neutrophil phagocytic ability in neutrophils isolated from blood of cecal ligation and puncture (CLP)-induced septic mice [62,63]. Further evidence to support this hypothesis comes from work showing that FPR2 activation in human neutrophils increased neutrophil phagocytic ability by promoting the expression of FcγR1 on neutrophils [64]. After the increased phagocytosis, there is evidence that SPMs (RvE1 and PD1) promote phagocytosis-induced apoptosis in neutrophils [26]. The mechanism for this effect appears to be by reducing neutrophil elastase and proteinase 3 release [52]. These reports suggest that certain SPMs promote host defense by increasing neutrophil phagocytic ability as well as augmenting later resolution by increasing neutrophil apoptosis.

LxA4 decreased neutrophil migration and reduced bacterial biofilm in a model of periodontitis caused by the *Porphyromonas gingivalis* bacterium [65]. This bacterium lays down biofilm on tooth and surrounding surfaces, which then initiates an inflammatory response, especially of neutrophils, which then migrate to the site of infection [65]. There was a clinical trial examining the use of a stable LxA4 analog in periodontitis. In this trial, 1 µM of LxA4 analog mouthwash reduced the amount of gingivitis plaque and bleeding over the 28-day time course of the study [66], providing clinical evidence that an SPM can be beneficial in resolving infectious inflammation. In this particular setting, it appears that inhibiting chronic host defense activation was beneficial.

NETs are traps formed from the release of nuclear chromatin into the extracellular environment. These traps contain large quantities of bactericidal protein granules, which contribute to the bacterial killing actions of neutrophils. The proteins extruded by neutrophils that make NETs bactericidal are histones, primary and secondary granules such as myeloperoxidase, elastase, gelatinase, cathepsin G, and proteinase 3. NETs formation is an important process in bacterial clearance [41,67]. On the other hand, high levels of NETs are associated with exacerbation of LPS-induced lung injury [68], formation of thrombi in murine sepsis [69], increased severity in patients with acute respiratory disease (ARDS) [70], and is a major mediator of death in sepsis [71]. Preincubation of mouse bone marrow neutrophils with LxA4 was shown to reduce NETs formation [70], while RvD4 decreased NETs formation in ionomycin-stimulated human neutrophils [72]. Therefore, the overall evidence shows that SPMs reduce NETs formation.

In a study of *P. aeruginosa* chronic lung infection, the authors administered RvD1 5 days after bacterial inoculation, near the peak of infection. Under these conditions, RvD1 reduced bacterial load, neutrophil infiltration, and lung injury [32]. A major mechanism of host defense in this setting was the increased phagocytosis of bacteria by neutrophils and macrophages to reduce bacterial burden. A similar result was obtained in a model of chronic *P. aeruginosa* infection in cystic fibrosis, where once again RvD1 reduced bacterial titer, neutrophil lung infiltration, and pulmonary tissue damage [73].

A report that showed a negative impact of SPM use was in a *pneumonia* model where the authors showed that use of LxA4 before administration of the pathogen was deleterious [74]. As LxA4 is one of the earliest SPMs produced [7], it is possible that these detrimental effects of LxA4 were due to its inhibitory effects on neutrophil action such that very early LxA4 administration may leave the host unable to mount an appropriate response. Taken together, all of these reports suggest that SPMs have dual contrasting effects on neutrophils during infection where they increase phagocytosis and help in bacterial clearance, but they also reduce neutrophil infiltration and promote apoptosis which help reduce acute inflammation. The precise mechanism by which an SPM can elicit such contrasting effects on neutrophils has not been fully elucidated. However, SPMs act on neutrophils to help clear pathogens as well as resolve infectious inflammation.

## 6. SPM Effects on Macrophage Activity

Resident tissue macrophages and monocyte-derived macrophages are involved in both phagocytosis of bacteria and the inflammatory response in host defense during bacterial infection [12]. These cells are also important in infection resolution, as they phagocytose apoptotic neutrophils in the process of efferocytosis. SPMs such as LxA4 have direct effects on monocytes to increase migration [75]. LxA4 also has direct effects on monocyte myofilaments to promote phagocytic ability [76,77]. Orally administered RvD1 down-regulated peritoneal macrophage genes involved in the transcription of mediators that regulate the reduction of macrophage phagocytosis [78]. These studies strongly indicate that SPMs promote monocyte/macrophage activity as part of host defense.

Cecal ligation and puncture (CLP) is a commonly used rodent model of polymicrobial sepsis [79]. The major advantage of this model is that it mimics important characteristics of human sepsis, such as being caused by bacterial infection and having an immunosuppressive phase [79,80]. Therefore, it is thought to be a better model for sepsis than the older LPS-injected models as the latter do not show an immunosuppressive phase nor are they caused by bacterial infection [81]. In this model, immunosuppression was well studied with respect to macrophages where these cells have lowered responsiveness to LPS [82,83]. LxA4 and RvD2 were able to reduce bacteria load and blood cytokine levels and increase survival in the CLP model of sepsis [38,39,84]. RvD2 acts specifically on its cognate receptor GPR-18 to promote macrophage phagocytic ability and efferocytosis of apoptotic neutrophils [85]. Furthermore, these effects of RvD2 on macrophage activity were mediated by enhancement of protein kinase A and Stat3 [39]. These reports suggest that in this model of sepsis, early administration of an SPM is beneficial. It is plausible that the mechanism for these beneficial effects in this model is through the actions of SPMs on neutrophils and macrophages to promote phagocytic ability and increase bacterial clearance [64,65].

Along these lines, peritoneal macrophages were reported to have reduced NF-κB expression in LxA4-treated CLP mice [38], and Mar1 reduced NF-kB activity in peripheral blood monocytes [86]. In vitro studies showed that RvD2 reduced TLR4 expression in macrophages stimulated with LPS [87]. Both these reports suggest that a possible mechanism for the reduced inflammatory response seen after SPM administration was due to reduced TLR4-NF-κB signaling in macrophages.

In human periodontitis, macrophages appeared to reduce phagocytic ability, which was restored by RvE1 administration [88]. Similarly, MCTR1 and MCTR2 increased phagocytosis of bacteria in human macrophages [23]. In further work, MCTR3, PCTR3 and RCTR3 all enhanced human macrophage phagocytosis of *E. coli* and IL-10 secretion [24]. These actions were mediated by tumor necrosis factor receptor-associated factor-3 (TRAF-3). In germ-free mice, LxA4 production was reported to be a key regulator of the anti-inflammatory cytokine IL-10 production, which was essential for mouse hyporesponsiveness to ischemia-reperfusion injury [89]. In bacterial sepsis however, overproduction of IL-10 is immunosuppressive, implicated as a cause of monocyte hyporesponsiveness to stimulation, and is deleterious in septic patients [90,91,92]. Interestingly, use of RvD2 and LxA4 in in vivo models of sepsis did not cause significant increase in systemic IL-10 [38,84]. So, use of SPMs in sepsis is generally regarded not to be immunosuppressive. The reason for the discrepancy in its effects on IL-10 are unclear but may be caused by the overall reduction in bacteria load, which then reduces the amount of total inflammatory response. To the best of our knowledge, there are no studies in vivo which report that there was an increase in monocyte/macrophage production of inflammatory mediators. The overwhelming body of evidence shows that macrophages are stimulated by SPMs as part of host defense to clear the invading pathogen without an increase in inflammatory mediator release.

## 7. SPM Action on T and B-Cells

Apart from their action on the innate immune system, there is evidence that SPMs also act to reduce cellular activity in the adaptive immune system. Specifically, there is evidence that RvE1 causes apoptosis of stimulated T-cells, while Protectin D1 decreases T-cell migration [93,94]. Additionally, RvD1, RvD2 and Mar1 decrease differentiation of naïve CD4 cells into Th1 and Th17 cells (inflammatory T-cells) and potentiate the production of T-regulatory (Treg; anti-inflammatory) cells [95]. These Treg cells were shown to down-regulate the adaptive immune system [96]. RvE1 was also reported to decrease Th17 and dendritic cell activation [97]. LxA4 reduced CD4 and CD8 T-cell migration into the central nervous system (CNS) of a mouse model of multiple sclerosis [98]. Furthermore, LxA4 reduced production of IL-17, TNFα and IFN-γ from T-cells taken from patients with relapse-remitting multiple sclerosis [98]. Similarly, Mar1 increased the ratio of Treg/Th1 in an ex vivo study using naïve CD4 T-cells obtained from patients with rheumatoid arthritis [99]. In a study of T-cells taken from congestive heart failure patients, Chiurchiu and coworkers reported that RvD1 and RvD2 did not affect proinflammatory cytokine release and that the lack of response was due to a reduction in the expression of the GPCR32 RvD1 receptor [100]. These studies strongly suggest that SPMs can down-regulate the T-cell adaptive immune system and provide benefit to a number of inflammatory diseases.

In *Mycobacterium tuberculosis,* an infection which is a slow, chronic disease with long latent infective periods, results with LxA4 are contrary to that obtained with SPMs in other bacterial infection models. In these studies, 5-LOX-deficient mice, with low LxA4, cleared infection efficiently and had increased survival [101]. Wild-type mice had significantly greater bacterial load compared to the 5-LOX-deficient mice after more than 21 days, suggesting that possible down-regulation of T-cell adaptive immunity by LxA4 is detrimental for host defense in this model. Furthermore, patients with symptomatic tuberculosis had greater levels of RvD1 and RvD2 than that of uninfected controls, seemingly supporting the hypothesis that there may be a failure to resolve infection.

SPMs also act on antibody-mediated immunity. RvD1 differentiated human B-cells into an antibody-secreting phenotype without affecting B-cell proliferation [102]. This resulted in greater IgG and IgM production. On the other hand, RvD1 decreased B-cell production of IgE, which is the major mediator in allergic disorders such as asthma and urticaria [103]. In a separate study, RvD1 decreased B-cell production of IgE in asthmatic patients, but this effect was not evident in patients who were taking corticosteroids [104]. On the other hand, LxA4 decreased antibody (IgG, IgM) production and reduced expansion of human memory B-cells [105]. These results suggest an SPM-specific action on B-cells.

Therefore, there is enough evidence to suggest that SPMs have the overall effect of downregulating T-cell activity of the adaptive immune system of host defense. On the other hand, SPMs appear to upregulate B-cell-mediated IgG production while inhibiting IgE production, providing evidence that SPMs have differing effects on the two different arms of adaptive immunity.

## 8. Quorum Sensing and Bacterial Virulence

**Figure 2:**
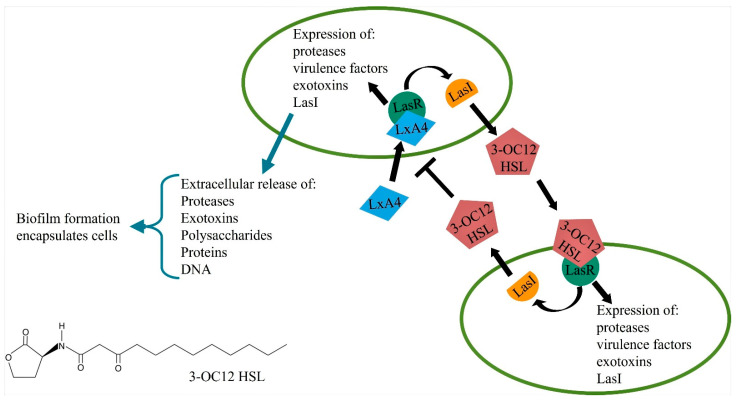
Bacteria such as *Pseudomonas aeruginosa* activate a quorum sensing signaling pathway when their population density reaches a particular threshold. In LasI/LasR pathway, LasI signaling increases production of quorum sensing inducer, N-3-oxododecanoyl-homoserine lactone (3—OC12-HSL). 3—OC12-HSL binds to its cognate receptor LasR, and then increases expression of virulence genes. This promotes release of exotoxins and proteases, and increases antibiotic resistance as well as biofilm formation, all of which augments virulence. LxA4 binds to and inhibits LasR receptor, which reduces *Pseudomonas aeruginosa* virulence.

Bacterial virulence is regulated by a complex signaling network called quorum sensing (QS) [106,107]. When the population density of many species of bacteria reaches a particular threshold, the QS network is activated. The most studied QS signaling pathway is in the gram-negative bacteria *P. aeruginosa* where there are 4 major interconnected signaling pathways [106]. Signaling molecules for these pathways are acyl homoserine lactones: N-(3-oxododeconoyl)-L-homoserine lactone (3-OC12-HSL) for the *las* pathway, N-butanoyl-L-homoserine lactone (BHL) for the *rhl* pathway, 2-heptyl-3-hydroxy-4-quinolone for the *Pseudomonas* Quinolone Signal (PQS) pathway, and 2-(2-hydroxyphenyl)-thiazole-4-carbaldehyde for the Integrated Quorum Sensing (IQS) pathway. The *P. aeruginosa* bacteria secrete these molecules, which then act on their cognate receptors to trigger coordinated expression of genes that regulate virulence [106,107]. In terms of hierarchy, the 3-OC12-HSL-Las pathway sits upstream of all the other pathways. Gram-positive bacteria utilize oligopeptides instead of acyl homoserine lactones. Another QS molecule is termed autoinducer-2 (AI-2), which is a boron-furan derivative and released by both gram-negative and gram-positive bacteria. The genes regulated by these pathways include but are not limited to the secretion of exotoxins, expression of antibiotic resistance genes (ARGs), elastase expression, and biofilm formation (Figure 2).

The increased release of exotoxin damages adjacent cells/tissues. Biofilm consists primarily of exopolysaccharides, nucleic acids, and proteins. Biofilms essentially encapsulate the bacteria, shielding it from host leukocytes and antibiotics. With the ever-increasing use of antibiotics, antibiotic resistance is a major problem with bacterial infections, and targeting the quorum sensing pathways may be an alternative [108,109].

Interestingly, molecules involved with QS signaling not only interact with bacteria but also interact with host cells. For instance, 3—OC12-HSL release from bacteria activates neutrophils, macrophages, fibroblasts, mast cells and B-lymphocytes [110]. The general outcome of these interactions is variable and has not been fully elucidated, where there are reports of it being proinflammatory, while there are other reports of it being immunosuppressive. It is speculated that the immunosuppressive effect may lead to an inability to clear pathogen and prolong infection. The variability in outcomes is thought to be concentration dependent. Overall, the activation of the QS mechanism is deleterious, as it either reduces the ability of the host to clear pathogen or overstimulates host response to cause tissue injury.

Taken together, the quorum sensing mechanism of virulence with its interaction with host defense makes it an attractive target for antimicrobials. Unlike traditional antibiotics, an anti-QS agent would attenuate virulence without significant growth inhibition or killing. So, one could envision that such an inhibitor could be used to lower virulence and allow host’s innate immunity to clear the pathogen. Such an inhibitor could also be used in conjunction with an antibiotic to increase the antibiotic’s efficacy without the problem of antibiotic resistance.

Interaction of SPMs and QS signaling: Recently there were reports that LxA4 can directly bind to and inhibit the LasR receptor in *P. aeruginosa* to reduce production of the exotoxin pyocyanin [67]. In further studies, LxA4 alone was directly able to reduce *P. aeruginosa* biofilm formation and increase ciprofloxacin efficacy to kill bacteria within the biofilm [111]. The mechanism of this action appeared to be a direct inhibition of *P. aeruginosa* virulence gene expression. These reports provide early evidence that an SPM can directly decrease bacterial virulence. These actions, which work directly on bacteria, support the notion that SPMs can work in tandem with the host, promoting host defense mechanisms and acting directly on bacteria to decrease their virulence. Such a mechanism would lower the burden of the host defense to clear the invading pathogen.

## 9. Concluding Remarks

**Figure 3:**
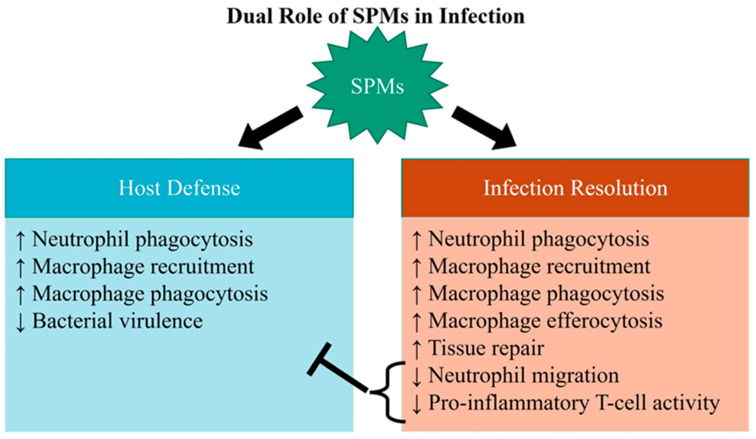
SPMs resolve inflammation as well as augment host defense. SPMs do however decrease neutrophil migration and decrease proinflammatory T-cell activity, which may attenuate host defense.

There is strong evidence that SPMs upregulate early host defense mechanisms to clear invading pathogens. These mechanisms include increased neutrophil phagocytosis as well as monocyte/macrophage recruitment and phagocytosis (Figure 3). In addition, there is evidence that certain SPMs have a direct action in reducing bacterial virulence. These actions are distinct from certain proresolution actions, such as reducing neutrophil migration and decreasing proinflammatory T-cell activity, both of which serve to attenuate tissue injury, but also suppress host defense. Therefore, reports support the hypothesis that SPMs activate host defense to help fight bacterial infection. Tissue and cellular mechanisms of how SPMs regulate this increase in host defense remain to be fully elucidated. Furthermore, the question of whether SPMs can be beneficial later in chronic infections where there may be T-cell (adaptive immune system) involvement is not yet answered.
